# L-Lysine α-Oxidase: Enzyme with Anticancer Properties

**DOI:** 10.3390/ph14111070

**Published:** 2021-10-22

**Authors:** Elena V. Lukasheva, Gulalek Babayeva, Saida Sh. Karshieva, Dmitry D. Zhdanov, Vadim S. Pokrovsky

**Affiliations:** 1 Department of Biochemistry, Peoples’ Friendship University of Russia (RUDN University), Miklukho—Maklaya Street 6, 117198 Moscow, Russia; elukasheva@yandex.ru (E.V.L.); babaevagulyalek@gmail.com (G.B.); 2 Laboratory of Combined Treatment, N.N. Blokhin Cancer Research Center, Kashirskoe Shosse 24, 115478 Moscow, Russia; skarshieva@gmail.com; 3 Institute of Biomedical Chemistry, Pogodinskaya Street 10/8, 119121 Moscow, Russia; zhdanovdd@gmail.com; 4 Center of Genetics and Life Sciences, Sirius University of Science and Technology, Federal Territory Sirius, 1 Olimpiisky Prospect, 354340 Sochi, Russia

**Keywords:** anticancer enzymes, tumor therapy, L-amino acid oxidase, L-lysine α-oxidase, L-lysine, colon cancer treatment

## Abstract

L-lysine α-oxidase (LO), one of L-amino acid oxidases, deaminates L-lysine with the yield of H_2_O_2_, ammonia, and α-keto-ε-aminocaproate. Multiple in vitro and in vivo studies have reported cytotoxic, antitumor, antimetastatic, and antitumor activity of LO. Unlike asparaginase, LO has a dual mechanism of action: depletion of L-lysine and formation of H_2_O_2_, both targeting tumor growth. Prominent results were obtained on murine and human tumor models, including human colon cancer xenografts HCT 116, LS174T, and T47D with maximum T/C 12, 37, and 36%, respectively. The data obtained from human cancer xenografts in immunodeficient mice confirm the potential of LO as an agent for colon cancer treatment. In this review, we discuss recently discovered molecular mechanisms of biological action and the potential of LO as anticancer enzyme.

## 1. Introduction

Cancer cells overconsume nutrients to satisfy energetic and biosynthetic needs for growth due to increased anabolic processes. Therapeutic strategy aimed at reducing the serum level of certain amino acids, and for that reason, the decrease of their intake by cancer cells has been extensively investigated during the last 40 years. Asparagine, arginine, and methionine were appointed as essential amino acids for cancer cells, and enzymes cleaving the aforementioned amino acids were reported to produce clinical effects against leukemia and solid cancers in animal experiments and clinical trials [1,2,3,4,5,6,7,8,9,10].

**Figure 1:**
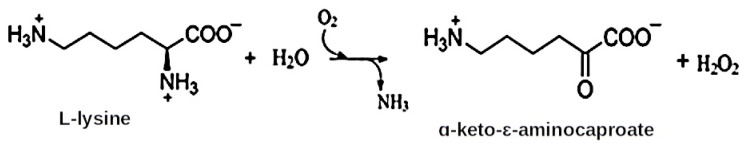
Scheme of the reaction catalyzed by L-lysine α-oxidase.

The natural oxidation of amino acid was discovered in 1910 [11]. Amino acid degrading oxidases, such as L-amino acid oxidases (LAAOs), were first described by Zeller and Maritz [12] and later isolated from both eukaryotic and prokaryotic cells [13,14,15,16,17,18]. LAAOs derived from snake venom are the most extensively investigated [19]. LAAOs, mainly FAD-containing enzymes, are homodimeric glycoproteins with a molecular mass of each monomer of about 50–70 kDa [20]. L-lysine α-oxidases (LOs, EC 1.4.3.14) represent distinct group of LAAOs with unique substrate specificity catalyzing the oxidative deamination of L-lysine with the formation of α-keto-ε-aminocaproate, ammonia, and H_2_O_2_ (Figure 1). Numerous biological effects of LO, including antimicrobial, antiviral, anti-protozoa, cytotoxic, antitumor, and anti-metastatic, have been reported over the last 40 years [21,22,23,24,25,26,27].

The aim of this review is to summarize the available data on the mechanisms of biological action, pharmacological data, as well as evaluation of LO as anticancer agent.

## 2. Structure of L-Lysine α-Oxidase from Different Sources

The structure of LOs was studied by X-ray diffraction analysis. The enzymes were obtained in crystal form from *Trichoderma viride (Tr. viride)* expressed in *Streptomyces lividans* TK24 and *Escherichia coli (E. coli)* [28,29,30]. LO from *Tr. viride* was expressed as a precursor (prLO) and the mature protein was shaped by cleavage of the N-terminal 77 residues [31]. The studied LOs are only enzymatically active as homodimers with molecular mass of 100–120 kDa (Table 1).

Each monomer includes 540 amino acid residues and contains three domains: FAD binding domain, substrate binding domain, and helical domain [28,29]. The FAD binding domain contains easily identifiable eight α-helices and nine β-chains, the substrate binding domain contains four α-helices and fourteen β-chains, and the helical domain contains seven α-helices. The domains are connected by a loop containing 388–391 amino acid residues. L-lysine is bound at the end of the funnel formed between the substrate binding domain, the helical domain, and the isoalloxazine ring of FAD at the bottom. The studied recombinant LOs showed highly homologous structures [28,29,30]. 

The carboxyl group of substrate forms’ hydrogen binds with R68 and Y369. The long and narrow hole for the substrate side chain is formed by hydrophobic side chains of F216, W371, F439, A475, and W476. The substrate binding induces conformational changes and its side chain takes an extended conformation. The α-amino group hydrogen binds to a carbonyl oxygen of A475 and forms cation–pi interaction with the indole ring of W476. These interactions are conserved in other LAAOs, such as LAAO from *Calloselasma rhodostoma* and LAAO from *Rhodococcus opacus* [28]. The side chain amino (ε-amino) group of L-lysine interacts with the side chain carboxy group of D212 and two water molecules, which are bound with the carbonyl oxygen of A440 and side carboxy group of D315. Triangular interaction of protonated L-lysine is important for achievement of high specificity to substrate. Substrate attachment in the active center leads to several conformational changes in the side chains of LO. One of them is the movement of W371 towards L-lysine to interact with its aliphatic part. This event blocks the substrate in the active center [28]. The binding site of LO expressed in *E. coli* is similar to that of LO of *Tr. viride* and *Streptomyces lividans* TK24. The L-lysine side chain is recognized by hydrophobic and hydrophilic amino acids (F293, W448, F516, A552, W553, D289) and is bound with two water molecules and carbonyl oxygen A517 [30]. Similarly in the other LAAOs, FAD interacts with the LO protein part through the atoms of the polypeptide chain [29]. During the reaction, FAD is reduced and α-imino acids are released as the products of LAAO reaction. FAD is oxidized by oxygen. Imino acids react with water with an α-keto acid and ammonia formation. As the catalytic reaction includes several steps, the total scheme is rather complicated; the proposed mechanism of action of LO is similar to those reported earlier for LAAO from *Calloselasma rhodostoma* [38]. LOs more intensively oxidize L-lysine compared to other positively charged amino acids. The higher affinity of LO towards L-lysine is congruent with significantly lower Km values (Table 1).

## 3. Pharmacokinetic Properties and Tissue Distribution of L-Lysine α-Oxidase

The evaluation of the pharmacokinetic parameters of protein substances allows us to assess their biological activity in the bloodstream, which is important, since even partial denaturation of enzymes can lead to its loss. Unfortunately, there are few investigations devoted to the pharmacokinetics study of the LO from different sources. T_1/2_ of LO from *Tr. viride* Y244-2 has been determined to be approximately 2 h, when it was intraperitoneally (*i.p*.) administered at a dose of 300 U/kg [39]. It was also shown that the plasma pharmacokinetics of LO from *Trichoderma* cf. *aureoviride* Rifai VKM F-4268D is characterized by a nonlinear curve. The decrease in the concentration of LO in the blood occurred independently of its dose and had a pronounced two-phase character: in the initial phase (mixed phase of distribution and elimination), the concentration of LO decreased faster than in the final (elimination) phase. LO had a short half-life in the bloodstream (T_1/2_ 0.86–1.23 h) when it was administered intravenously (*i.v.*) to mice. T_1/2_ was longer at lower doses (1–1.5 mg/kg) and shorter at a higher dose (3 mg/kg) [40]. A similar decrease of T_1/2_ with raising doses was observed for other enzymes [41]. The pharmacokinetics of LO after repeated *i.v.* administration did not differ from the ones after a single administration. It was also found that LO accumulates in various organs, including the brain, and remains for a long period of time (T_1/2_ for liver 26.10 ± 2.60 h, spleen 26.05 ± 3.23 h, kidney 12.00 ± 0.79 h, brain 9.41 ± 1.10 h, heart 7.75 ± 0.73 h) [40,42]. Thus, the values of plasma clearance, the elimination rate constants, as well as the relatively short half-life of the enzyme in blood plasma indicate that LO is rapidly removed from the blood. However, these pharmacokinetic parameters of LO are comparable to the data for *i.v.* administration of native L-asparaginase from *E. coli* to mice [43].

## 4. L-Lysine Depletion by L-Lysine α-Oxidase In Vitro and In Vivo

Cancer cells are more sensitive to the deficiency of essential growth factors, including amino acids. Reduction of L-lysine level is the primary mechanism contributing to the anticancer effect of LO.

Reduction of L-lysine level in vitro. After LO from *Tr. viride* Y244-2 (10 mU/mL) incubation for 2 h in RPMI 1640 medium, L-lysine was eliminated completely. When LO plummeted to 1 mU/mL, the concentration of L-lysine decreased by 40% after 2 h [39].

Reduction of L-lysine level in vivo. Reiken et al. reported that a 70% L-lysine reduction in blood is sufficient to suppress the growth of tumor cells in mice. Unlike normal cells, cancer cells are sensitive to such decrease [44]. LO from *Trichoderma* cf. *aureoviride* Rifai VKM F-4268D reduced the concentration of L-lysine in different organs, including the brain. An *i.v.* LO administration to mice at a dose 1 mg/kg caused a gradual decrease of L-lysine concentration by 20% after 1 h, and by 60% after 6 h [45].

The base level of L-lysine in murine plasma is 135.8 ± 16.2 µm [40]. Only 25% of L-lysine was found 15 min after a single *i.v*. injection of 1.6 mg/kg LO. L-lysine was almost entirely removed after 1 h and its concentration remained at negligible level for 9 h, then gradually recovered by 24 h [39,40,46]. One hour after a single *i.p*. LO administration at a dose of 30 U/kg, the concentration of L-lysine decreased by 15%. The concentration of L-lysine did not return to the initial value 24 h after *i.p.* injection, remaining as 50% of the base level.

Both in vitro and animal experiments have shown that LO is an effective enzyme for reducing L-lysine concentration.

## 5. Mechanisms of the Cytotoxic Action of L-Lysine α-Oxidase

The mechanism of LO cytotoxic action is associated with the depletion of L-lysine as well as accumulation of reaction products [13].

The cytotoxic effect is enhanced by exposure of LAAOs on the cell membrane, since cancer cells have higher concentrations of lipids than normal cells [47]. Carbohydrate moieties of the molecule were proposed to be important for the binding of the enzyme to the cell surface and its cytotoxic effect [48]. A distinct channel for H_2_O_2_ release was found in the crystal structure of LAAO from *Calloselasma rhodostoma*. The channel is located near the glycosylation site on N172. This structural arrangement may explain the mechanism of apoptosis, since the enzyme is attached to the cell surface via a glycan fragment [31]. H. Ande and coauthors attempted to evaluate the impact of the carbohydrate part of LAAO on the penetration of the enzyme into cell and cytotoxic effect on the Jurkat cell line. However, the cytotoxic properties of the enzyme did not change after de-glycosylation [49]. Despite the lack of evidence of intracellular penetration of LO, a significant increase in the intracellular concentrations of reactive oxygen species (ROS), which lead to cell death, was reported in PC-12 cells [50]. ROS and H_2_O_2_ are known to have direct cytotoxic effect and can modify signaling proteins and membrane lipids [51]. As ROS exhibit a wide range of intracellular effects, they can also affect nucleic acid synthesis.

The cytotoxic effect of LO from *Tr. viride* Y244-2 decreased when catalase was added to the medium [39]. Antioxidants also play an important role in the reduction of cytotoxic effects. The thioredoxin reductase (TXNRD) inhibitor auranofin enhanced the cytotoxic effect of LO in transformed breast cancer epithelial cells. Cell damage was directly associated with a decrease of glutathione level, which also increased the intracellular level of ROS. LO induces caspase-dependent cell death, via both internal and external apoptosis pathways. Activation of caspases is associated with enhanced permeability of inner mitochondrial membrane caused by oxidative stress. However, other researchers have shown that LAAO does not cause caspase-dependent cell death and zVAD-fmk caspase inhibitor did not suppress cytotoxicity [15,52].

Various signaling pathways have been reported to contribute to the cytotoxic effect of LAAOs. Released H_2_O_2_ inhibits the growth of HepG2 cells via the Tumor Growth Factor-β (TGF-β) signaling pathway. The morphological changes caused by LAAO from *Agkistrodon blomhoffii ussurensis* were less pronounced after treatment with catalase and an inhibitor of TGF-β LDN-193189 [53]. This effect may be related to the activation of Cyr61, which promotes the proliferation of tumor cells and inhibits apoptosis by depressing the expression of p53 in the TGF-β signaling pathway.

Cell death is suppressed by inhibitors of necroptosis and ferroptosis. Necrostatin-1 (Ncr-1) and ferrostatin-1 (Fer-1) decreased cytotoxicity of LO. The combination of LO with auranofin enhanced LO-induced necroptosis and ferroptosis by ROS-dependent mechanisms. The main regulator of antioxidant response nuclear factor erythroid 2-related factor 2 (NRF2) was induced with LO and this contributes to the survival of cells under oxidative stress [15,52,54].

Cancer cells overexpress anti-apoptotic proteins, especially the Bcl-2 family, to overcome stress signals. LAAO reduced Bcl-2 expression in HepG2, HL-60, SW480, and SW620 cells and bolstered the expression of proapoptotic BID, FADD, and miR-16 genes in K562 cells [55,56].

Tumor tissues are characterized by increased concentration of polyamines (PA). Reduction of PA levels is considered as a possible contribution to the anticancer effect. It was shown in Balb/c mice that LO reduced the concentration of L-arginine and L-ornithine. Since these amino acids are precursors of PA, LO may significantly reduce the concentrations of putrescine, spermine, and spermidine, offering a new basis for the anticancer activity of LO [45].

## 6. The Cytotoxic Effects of L-Lysine α-Oxidase In Vitro

A wide range of cells of squamous and glandular origin are sensitive to LAAOs [23,50,51,57,58,59,60,61,62,63,64,65,66]. LAAO of fungal origin, *Amanita phalloides* and *Clitocybe geotropa*, showed cytotoxic activity against Jurkat T-lymphoblastic leukemia and human breast epithelial MCF7 cells cultures and the most pronounced effect was in Jurkat cells (Table S1, Supplementary Materials) [15]. Colon cancer cell lines were reported to be among the more sensitive ones.

For the LAAO of the snake *Cryptelytrops purpureomaculatus* venom (L-leucine specific), a significant cytotoxic effect was demonstrated against primary and metastatic colon cancer cells SW480 and SW620, with an IC_50_ of 29.43 and 23.19 µg/mL, respectively [54]. At the same time, the IC_50_ for colon tumor cells CD-18co was lower (15.99 µg/mL).

LO from *Trichoderma* cf. *aureoviride* Rifai VKM F-4268D showed a dose-dependent cytotoxic effect towards different cancer cell lines (Table 2). The cells of erythromyeloblastic leukemia K562, breast cancer MCF7, and colon cancer LS174T with IC_50_ 3.2 × 10^−8^, 8.4 × 10^−7^, and 5.6 × 10^−7^ mg/mL were the most sensitive to LO from this source [23].

The sensitivity of cell lines to LO may be related to the difference in the dependence of cells on L-lysine concentration and oxidative stress.

## 7. The Antitumor Effects of L-Lysine α-Oxidase In Vivo

In transplanted human and murine solid tumors in mice, LAAOs showed a wide spectrum of antitumor activity. LAAO from the snake venom *Ophiophagus hannah* effectively suppressed the growth of PC-3 prostate cancer xenografts [51]. LAAO from *Agkistrodon acutus* inhibited the growth of hepatoma 22, sarcoma 180, and ascitic Ehrlich carcinoma, depending on dose. At a dose of 6 mg/kg, the growth inhibition was 40.5, 42.4, and 43.4%, respectively [67]. The spectrum of tumors sensitive to LO from *Trichoderma harzianum* Rifai and *Tr. viride* Y244-2 is represented by solid tumors of different species. The most sensitive murine transplantable tumors were breast adenocarcinoma Ca755 (TDI 96%), ascitic hepatoma 22 (T/C 201%), melanoma B16, colon carcinoma AKATOL, and cervical cancer RSHM5 (TGI 81, 75, and 79%), respectively [68]. Intraperitoneal administration of 70 U/kg of LO increased the lifespan by 34–48% (Table 3) [39]. Two human colon cancer xenografts, HCT116 and LS174T, and breast adenocarcinoma T47D implanted subcutaneously into Balb/c nude mice showed high sensitivity to LO with a T/C of 12, 37, and 36%, respectively (*p* < 0.05) (Table 4) [23].

These findings suggest that LO may be considered as an effective anticancer agent for the treatment of solid tumors in vivo.

## 8. Immunogenicity of L-Lysine α-Oxidase

The potential immunogenicity of many protein substances, in particular enzymes, is a limiting factor for clinical use. An immune response with the formation of antibodies occurs a few days after a single injection of proteins. Repeated administration of proteins can increase their immunogenicity and the rate of neutralization by antibodies in the blood; as a result, the efficacy of drugs will decrease. Therefore, the detection of anti-drug antibodies in patients is a necessary requirement in clinical trials for the approval of protein-based drugs. The effect of LO from *Trichoderma harzianum* Rifai on the immune response to ovalbumin and L-asparaginase from *E. coli*, respectively, was studied. It has been shown that the level of antibodies in animals after a single administration of ovalbumin at a dose of 2 mg/kg, followed by the injection of LO at a dose of 100 U/kg, was lower than in control animals without LO. Repeated administrations of LO did not affect the immune response against L-asparaginase, which is a stronger immunogen. In addition, plasma analysis of mice immunized with *i.v.* LO injection did not reveal an immune response to the T-dependent antigen. LO at a therapeutic dose or doubled dose did not significantly affect the leukocyte migration capacity compared to the control and had no suppressive effect on the delayed hypersensitivity reaction to xenogenous erythrocytes. It was also shown that LO at a dose of 35 U/kg, administered parenterally, did not suppress the functional activity of T-lymphocytes and did not have mitostatic activity, which is an indication in favor of the enzyme compared with other antitumor agents [69,70].

## 9. Research Areas for L-Lysine α-Oxidase as Anticancer Agent

LO isolated from different species have shown robust results against a number of tumors in vivo such as breast cancer, cervical cancer, melanoma, and colon carcinoma. The efficacy of LO strongly depends on the sensitivity of cells of different tumors to L-lysine deficiency and oxidative stress.

The data obtained from human cancer xenografts in immunodeficient mice confirm the potential of LO as an agent for colon cancer treatment. Colon cancer develops from rapidly dividing epithelial cells that have a high metabolic rate [71]. Colon cancer cells are auxotrophic cationic amino acids, especially L-arginine. The high demand for amino acids to support growth leads to increased regulation of amino acid transporters to meet these requirements [72,73]. It was reported that the expression of the SLC7A1 gene, which encodes the cationic amino acid transporter CAT1, is increased (by 70%) in colon cancer cells. The G0/G1 phase arrest of the cell cycle and the death of colon cancer cells Caco-2, SK-Co-1, SW837, and T84 were observed 48 h and 72 h after arginine deprivation. However, the L-citrulline addition restored the reduced level of argininosuccinate synthase-1 expression and significantly increased cell survival [74,75]. LO reduces the level not only of L-lysine, but also of L-arginine and L-ornithine [34], induces inhibition of cell viability, apoptosis in vitro, and suppression of tumor growth in vivo. Therefore, targeting cationic amino acids may be effective in cancer treatment [76].

Recently, it was determined that the EGFR (Epidermal Growth Factor Receptor)-mutant and EGFR-tyrosine kinase inhibitors resistant NSCLC (non-small cell lung cancer) cells, but not normal human lung fibroblasts, are sensitive to lysine deprivation. Moreover, the lysine reduction could enhance the cytostatic effect of osimertinib in EGFR-mutant NSCLC cells and it was connected with the regulation of the lysine catabolizing enzyme, α-aminoadipate aminotransferase by EGFR–AKT signaling [77].

In addition, low concentrations of lysine have been shown to inhibit the growth of various tumor cells, while high concentrations stimulate cell growth. For example, a four-fold decrease of lysine concentration relative to normal in the culture medium of SNU398 hepatocellular carcinoma cells had a minimal effect on cell growth. However, total lysine deprivation dramatically suppressed cell growth and colony formation. This was associated with the G0/G1 cell cycle arrest and induction of cell apoptosis [78]. Jang et al. showed that lysine deprivation inhibits mTORC1 (mammalian target of rapamycin complex 1) activity in NSCLC cell lines. Cell growth factors such as insulin or IGF-1 (insulin-like growth factor 1) were able to restore the decreased mTORC1 activity in cells cultured in serum-depleted media, but not in cells deprived of serum and lysine. The GCN2 (general control nonderepressible 2) kinase, which plays a key role in modulating the metabolism of amino acids in response to nutrient deficiency, and AMPK (adenosine monophosphate-activated protein kinase) were involved in the lysine deprivation-mediated inhibition of mTORC1 [79]. Lysine starvation has also been shown to inhibit the growth and proliferation of human breast cancer cells [52].

The promising approaches to improve the pharmacokinetics and increase the effectiveness of biological drugs include genetic manipulation, encapsulation, and protein conjugates. To increase the selectivity of the antitumor effect, methods for obtaining LO conjugated with antibodies against specific membrane proteins, such as the CD5 receptor, have been developed. These conjugates had a slightly reduced enzymatic activity of LO and immunological activity of antibodies [17,80]. ADI-PEG-20 is arginine deiminase conjugated to polyethylene glycol, which enhanced effect due to prolonged half-life and reduced immunogenicity. Clinical trials have been completed with ADI-PEG-20 for hepatocellular carcinoma [81,82] and melanoma [83]. Clinical trials of native asparaginase in patients with acute lymphoblastic leukemia (ALL) have shown high immunogenicity, hepatotoxicity, and toxicity to the pancreas [84,85,86]. The use of the PEGylated form significantly improved survival and reduced toxicity [87]. Currently, clinical trials are evaluating the safety and efficacy of erythrocyte-encapsulated asparaginase (GRASPA) not only for the treatment of ALL, but also for pancreatic cancer. Results show that adding the GRASPA to chemotherapy improves overall survival compared to monotherapy [88,89,90]. PEGylated L-asparaginase from *E. coli* (Oncospar) was approved by the FDA for the treatment of patients with acute lymphoblast leukemia (Table S2, Supplementary Materials) [91,92,93,94,95,96,97,98]. Currently, a clinical trial evaluating soft tissue sarcoma is in progress (NCT03449901) [99]. PEGylated methionine gamma-lyase from *Pseudomonas putida* (MGL) was found to have favorable kinetic properties and was more stable than the native enzyme. Both forms of the enzyme were studied in patients with breast, renal, and lung cancer and lymphomas [100,101]. The erythrocyte-encapsulated form of MGL was shown to have better pharmacokinetic properties and improved efficacy [102]. The use of advanced nanotechnologies and functional fillers for LO may improve the delivery system and pharmacokinetics, which offered new perspectives for the effective treatment of cancer.

## 10. Conclusions

In recent years, cancer chemotherapy aimed at the enzymatic cleavage of certain amino acids has developed significantly. One of the advantages of enzymes as drugs is the fact that in the body they, like all proteins, are metabolized to form non-toxic amino acids. Amino acid oxidases convert the alpha amino group in amino acids to keto acids. The review is devoted to a group of amino acid oxidases that are called LOs because they are specific towards L-lysine and, to some extent, to its structural analogs. LOs effectively reduce the level of L-lysine in vitro and in vivo. Experiments show that the principal mechanisms of the LOs anticancer effect are depletion of L-lysine, induction of oxidative stress, and a decrease in the concentration of polyamines in the body. 

In vitro a dose-dependent cytotoxic effect of LO towards different cancer cell lines was detected. In vivo a wider range of malignant tumors are sensitive to the action of LO in comparison with currently used asparaginases. The data obtained from human cancer xenografts in athymic mice confirm the potential of LO as an agent for colon cancer treatment. The prominent synergistic effect of LO combined with irinotecan or cisplatin entails and encourages further study of LO-containing combination therapy.

## Figures and Tables

**Table 1:** Biochemical properties of L-lysine α-oxidases from different microorganisms.

Sources of LO	Molecular Mass, кDa	pH Optimum	Substrate Specifity			K_m,_ mM	Reference
			Substrate	Relative Activity, %	Specific Activity, U/mg		
*Trichoderma viride* Y244–2	116	4.5–9.2	L-Lys	100	66	0.04	[32]
			L-Orn	18.2		0.44	
			L-Phe	8.3		14	
*Trichoderma* *harzianum* Rifai	100–120	4.5–10	L-Lys	100	40	0.014	[33]
			L-Orn	5.1		0.5	
			L-Arg	5.9		0.36	
*Trichoderma cf. aureoviride* Rifai VKM F-4268D	115–116	4.5–9.5	L-Lys	100	99	0.01	[34]
			L-Orn	8.3			
			L-Arg	5.8			
*Trichoderma viride* i4	110	8.0–9.0	L-Lys	100	90	0.026	[35]
			L-Orn	25		0.625	
			L-Arg	16		0.68	
*Trichoderma viride* (cloned to *Streptomyces lividans* TK 24)	116	ND	L-Lys	100	80	ND	[29]
			L-Arg	6.9			
			L-Orn	18.3			
			L-Phe	1.7			
			L-Tyr	1.4			
*Rhodococcus* sp. AIU Z-35-1	100	8	N^α^-Z-L-lys L-Lys	100 53	19.1 10.12	12.7 0.062	[36]
			L-Arg	61		0.42	
			L-Orn	88			
*Pseudomonas* sp. AIU 813	110	7	L-Lys	100	1.16	ND	[37]
			L-Orn	31			
			L-Arg	6			

ND—not determined.

**Table 2:** Cytotoxic and apoptotic effects of L-lysine α-oxidase isolated from fungus *Trichoderma*.

Sources of LO	Cell Line	IC_50_, mg/mL	Reference
*Trichoderma* cf. *aureoviride* Rifai VKM F-4268D	K562	3.2 × 10^−8^	[23]
	LS174T	5.6 × 10^−7^	
	HT29	8.2 × 10^−4^	
	SCOV3	9.9 × 10^−7^	
	PC3	2.6 × 10^−6^	
	MCF7	8.4 × 10^−7^	
	PC12	ND	[50]
*Trichoderma viride* Y244-2	L5178Y	1.5 × 10^−5^	[39]

ND—not determined.

**Table 3:** Antitumor activity of L-lysine α-oxidase in murine tumor models.

Sources of LO	Tumor Model	TGI, %	Range of Effective Doses, U/kg	Reference
*Trichoderma viride* Y244-2	L1210	ND	70	[39]
*Trichoderma harzianum* Rifai	Hepatoma 22 A	*	35–300	[68]
	Ca755	95	200–350	
	Melanoma B16	81	350	
	AKATOL	75	200–300	
	RSHM-5	79	200–300	
	Sarcoma 180	61	200–300	

TGI—Tumor Growth Inhibition; ND—not determined; * LO treatment increased life span substantially (201%), Complete Remission 29–66%.

**Table 4:** Antitumor activity of L-lysine α-oxidase in human xenografts.

Sources of LO	Tumor Model	T/C, %	Reference
*Trichoderma* cf. *aureoviride* Rifai VKM F-4268D	HCT116	12 *	[23]
	SKBR3	49	
	LS174T	37 *	
	Melanoma Bro	51	
	SKOV3	35	
	Hepatocellular carcinoma Alex	54	
	T47D	36 *	

T/C—Treatment/Control; Range of effective doses 75–150 U/kg; * *p* < 0.05.

## Data Availability

Data sharing not applicable.

## Supplementary Materials

The following are available online at www.mdpi.com/article/10.3390/ph14111070/s1, Table S1. Sensitive models to various of L-amino acid oxidases; Table S2. Results of clinical trials of amino acid degrading enzymes.
