# Supplementary material for: L-Lysine α-Oxidase: Enzyme with Anticancer Properties
Source: Pharmaceuticals (Basel). 2021 Oct 22;14(11):1070. doi: 10.3390/ph14111070 (PMC8618108; doi:10.3390/ph14111070)
Supplement: Supplementary file 1 [file pharmaceuticals-14-01070-s001.zip › pharmaceuticals-1378393-supplementary.pdf]

pharmaceuticals

Supplementary Materials

L-Lysine α-Oxidase: Enzyme with Anticancer Properties

Elena V. Lukasheva [1], Gulalek Babayeva [1,2], Saida Sh. Karshieva [2], Dmitry D. Zhdanov [3] and Vadim S. Pokrovsky [1,2,4,*]

1 Department of Biochemistry, Peoples' Friendship University of Russia (RUDN University), Miklukho—Maklaya Street 6, Moscow 117198, Russia; elukasheva@yandex.ru (E.V.L.); babaevagulyalek@gmail.com (G.B.)
2 Laboratory of Combined Treatment, N.N. Blokhin Cancer Research Center, Kashirskoe Shosse 24, Moscow 115478, Russia; skarshieva@gmail.com
3 Institute of Biomedical Chemistry, Pogodinskaya Street 10/8, Moscow 119121, Russia; zhdanovdd@gmail.com
4 Center of Genetics and Life Sciences, Sirius University of Science and Technology, Federal Territory Sirius, 1 Olimpiisky Prospect, Sochi 354340, Russia
* Correspondence: v.pokrovsky@ronc.ru

Citation: Lukasheva, E.V.; Babayeva, G.; Karshieva, S.Sh.; Zhdanov, D.D.; Pokrovsky, V.S. L-Lysine α-Oxidase: Enzyme with Anticancer Properties. *Pharmaceuticals* **2021**, *14*, 1070. https://doi.org/10.3390/ph14111070

Academic Editor: Alfredo Berzal-Herranz

Received: 22 September 2021
Accepted: 20 October 2021
Published: 22 October 2021

Publisher's Note: MDPI stays neutral with regard to jurisdictional claims in published maps and institutional affiliations.

Copyright: © 2021 by the authors. Licensee MDPI, Basel, Switzerland. This article is an open access article distributed under the terms and conditions of the Creative Commons Attribution (CC BY) license (http://creativecommons.org/licenses/by/4.0/).

Table S1. Sensitive models to various of L-amino acid oxidases.

Sources of LAAO	Name of LAAO	Cell type	IC_{50}, mg/ml	Reference
Amanita phalloides<br>Clitocybe geotropa	Ap-LAAO<br>Cg-LAAO	Jurkat<br>MCF7	ND<br>ND	[15]
Ophiophagus hannah	OH-LAAO	PC-3<br>B16F10<br>HT-1080	$5{\times}10^{-5}$<br>$1{,}7{\times}10^{-4}$<br>$6{\times}10^{-4}$	[51]<br>[66]
Cryptelytrops purpureomaculatus	CP-LAAO	SW480<br>SW620<br>CCD-18co	$2{,}9{\times}10^{-2}$<br>$2{,}3{\times}10^{-2}$<br>$1{,}5{\times}10^{-2}$	[54]
Lachesis muta	Lm-LAAO	MCF7<br>AGS	$1{,}4{\times}10^{-3}$<br>$22{,}7{\times}10^{-3}$	[57]
Bungarus fasciatus	BF-LAAO	A549	$1{,}2{\times}10^{-3}$	[58]
Bothrops leucurus	Bl-LAAO	LL 24<br>MKN-45<br>HUTU<br>RKO	ND	[59]
Agkistrodon acutus	ACTX-6<br>ACTX-8	A549<br>HeLa	$2{\times}10^{-2}$<br>ND	[64]
Bothrops atrox	Batrox-LAAO	HL-60<br>B16F10<br>PC12<br>Jurkat	$5{\times}10^{-2}$<br>$2{,}5{\times}10^{-2}$<br>ND<br>ND	[65]

ND—not determined.

Table S2. Results of clinical trials of amino acid degrading enzymes.

Enzyme	Indications	Results of clinical data	References
L-Asparaginase	Acute Lympho-blastic Leukemia	5-year survival rate 83%<br>Side Effects:<br>- Allergic reaction 15%<br>- Pancreatitis 7%<br>- Coagulopathy 4.5%	[84-86]
	T/NK-cell lym-phoma	5-year survival rate 55.6%<br>Side Effects:<br>- Leukopenia 22.2%<br>- Pancreatitis 5.6%<br>- Hyperglycemia 22.2%<br>- Allergic reaction 5.6%	[94]
PEG Asparagi-nase	Acute Lympho-blastic Leukemia	5-year survival rate 90.8%<br>Side Effects:<br>- Hypersensitivity 1.5%<br>- Osteonecrosis 3.5%<br>- Pancreatitis 2.8%	[87]
Asparaginase Encapsulated in Erythrocytes	Acute Lympho-blastic Leukemia	2-year overall survival 35.7%<br>Complete remission 70%<br>Side Effects:<br>- Hyperbilirubinemia 20.7%<br>- Deep vein thrombosis 7%	[88]
	Pancreatic Cancer	*6-month progression-free survival 20.4%	[89]

		*6-month overall survival 53.3%<br>*Side effects:<br>- Physical health deterioration 12.9%<br>- Neutropenia 12.9%<br>- Anemia 8.6%	
		Side effects:<br>- Immunogenicity 83.3%<br>- Gastrointestinal disorders 37.9%<br>- General disorders 18.2%	[90]
PEG-Arginase	Melanoma<br>Prostate<br>Adenocarcinoma	Complete remission >30 months<br>Side effects:<br>- Nausea<br>- Skin rash<br>- Diarrhea	[95]
	Hepatocellular Carcinoma	Overall survival (23.7 weeks) 91.3%<br>Progression-free survival (6 weeks) 95.7%<br>Side effects:<br>- Fatigue 59.3%<br>- Oedema 25.9%<br>- Anemia 7.4%<br>- Hyponatremia 11.1%	[96]
Pegylated Arginine Deiminase (ADI-PEG-20)	Melanoma	6-month progression-free survival (ASS negative) 60%<br>Side effects:<br>- Allergic reaction 2.5%<br>- Thrombocytopenia 2.5%<br>- Neutropenia 7.6%<br>- Anemia 2.5%	[97]
	Acute Myeloid Leukemia	6-month progression-free survival 30%<br>Side effects:<br>- Skin rash 9.3%<br>- Hyperuricemia 7%<br>- Leukopenia 7%<br>- Anemia 4.7%	[98]

* combined chemotherapy with gemcitabine.
